# Identification and classification of silks using infrared spectroscopy

**DOI:** 10.1242/jeb.128306

**Published:** 2015-10

**Authors:** Maxime Boulet-Audet, Fritz Vollrath, Chris Holland

**Affiliations:** 1 Department of Life Sciences, Imperial College London, London SW7 2AZ, UK; 2 Department of Zoology, University of Oxford, Oxford OX1 3PS, UK; 3 Department of Materials Science and Engineering, University of Sheffield, Sheffield S1 3JD, UK

**Keywords:** Silkworm, Cocoon, Lepidopteran, Multivariate analysis, Phylogenetic

## Abstract

Lepidopteran silks number in the thousands and display a vast diversity of structures, properties and industrial potential. To map this remarkable biochemical diversity, we present an identification and screening method based on the infrared spectra of native silk feedstock and cocoons. Multivariate analysis of over 1214 infrared spectra obtained from 35 species allowed us to group silks into distinct hierarchies and a classification that agrees well with current phylogenetic data and taxonomies. This approach also provides information on the relative content of sericin, calcium oxalate, phenolic compounds, poly-alanine and poly(alanine-glycine) β-sheets. It emerged that the domesticated mulberry silkmoth *Bombyx mori* represents an outlier compared with other silkmoth taxa in terms of spectral properties. Interestingly, *Epiphora bauhiniae* was found to contain the highest amount of β-sheets reported to date for any wild silkmoth. We conclude that our approach provides a new route to determine cocoon chemical composition and in turn a novel, biological as well as material, classification of silks.

## Abstract (precis)

**Summary:** FTIR analysis performed on unspun silks and cocoons from 35 different species provides information regarding the biochemical diversity and evolution of this group of materials.

## INTRODUCTION

Silkworm silk is a high-value agricultural product offering sustainable harvesting that directly contributes to poverty alleviation in rural communities (Astudillo et al., 2014; Dooley, 2004). Yet, it also has growing technical applications (Borkner et al., 2014; Omenetto and Kaplan, 2010). Developments in mulberry sericulture and the increasing use of fibres from ‘wild’ silkworms provide the backdrop for increased interest in understanding the diversity of all silks. Not surprisingly, millions of years of divergent evolution have resulted in a rich biodiversity of silks (Scoble, 1999). Typically used in cocoons, this class of materials consists of a silk fibroin protein thread of up to 1 km long coated with sericin proteins acting as a resin/matrix glue (Chen et al., 2010b). This non-woven composite structure (Chen et al., 2010a) regulates gas flow and humidity (Danks, 2004; Horrocks et al., 2013; Roy et al., 2012), as well as protecting the encased pupae from predation (Ishii et al., 1984), micro-organisms (Franceschi and Nakata, 2005) and the environment (Chen et al., 2012b). Silkworms produce cocoons with a broad variety of morphologies and architectures, ranging in porosity from loose meshes to full shells, with or without an exit opening (Chen et al., 2012c). Cocoons may also incorporate extraneous materials as well, such as integrated leaves for camouflage or an internally applied calcium oxalate solution that hardens the cocoon and may impart toxicity (Arnott and Webb, 2000; Chen et al., 2012c; Franceschi and Nakata, 2005; Gheysens et al., 2011; Takahashi et al., 1969; Teigler and Arnott, 1972b). The diversity of lepidopteran silk materials includes a molecular dimension, with amino acid analysis showing widely varying chemical compositions of silkworm silk (Hwang et al., 2001). However, while more advanced biochemical methods can inform on protein size (Hwang et al., 2001; Inoue et al., 2000; Mita et al., 1994), amino acid residue patterns (Navarro et al., 2008) and propensity to fold (Dicko et al., 2008), they are often labour intensive and expensive. Hence, only a handful of fibroin proteins have been sequenced to date (Tanaka and Mizuno, 2001). Furthermore, these methods are often focused on one specific molecular component of the cocoon and are unable to account for the other compounds present.

An alternative approach to achieve a broader assessment of chemical diversity is to employ complementary spectroscopic and scattering techniques (Gheysens et al., 2011; Warwicker, 1954). For example, the use of attenuated total reflection infrared spectroscopy (ATR-IR) is particularly well suited to studying silks in all forms as it is capable of measuring rough and deformable solids (Chen et al., 2012a; Gheysens et al., 2011), as well as turbid and concentrated protein solutions (Boulet-Audet et al., 2011). Requiring only minimal sample preparation, ATR-IR can selectively probe the inside or outside surface of silk cocoons, providing information on the local chemical composition (Boulet-Audet et al., 2014; Chen et al., 2012a, 2007). This spectroscopic method can determine (i) the level of protein crystallinity (Boulet-Audet et al., 2008), (ii) secondary structure (Goormaghtigh et al., 2006), and (iii) specific protein components such as sericin (Barth, 2000; Teramoto and Miyazawa, 2003). Infrared spectra are also indicative of (iv) non-protein molecules present in silk, such as the amount of water (Boulet-Audet et al., 2011), calcium oxalate (Chen et al., 2012b; Gheysens et al., 2011) and carbohydrate (Lu et al., 2011; Schulz and Baranska, 2007). In addition, the multivariate analysis of infrared spectra can (v) discriminate and classify samples based on their degree of relatedness. This infrared-based classification approach can even discriminate bacterial species (Kansiz et al., 1999; Preisner et al., 2007), types of human hairs (Panayiotou and Kokot, 1999), and coffee bean varieties (Briandet et al., 1996), as well as providing information for the construction of taxonomic trees (Zhao et al., 2006).

In this study, we analysed unspun native silk feedstock from six species across the Saturniini and Attacini tribes, and spun silks from 35 species across the Lepidoptera and Arachnida. Multivariate and hierarchical clustering analysis performed on over 1000 individual spectra allowed us to build taxonomic trees and compare them with trees based on protein-coding nuclear genes (Chen et al., 2012c; Regier et al., 2005, 2008a,b, 1998, 2002). As we demonstrate below, we identified several interesting outlier species that produce silk with very different chemical compositions and provide a hypothesis as to their origin. These newly characterised silks could even have greater potential for use in industrial and biomedical applications than those currently employed today.

## RESULTS

### Native feedstock spectral features

To evaluate the chemical variability of unspun silk without exogenous material, we used infrared spectroscopy to compare the native feedstocks of key species from the Lepidoptera genera *Actias*, *Attacus*, *Bombyx* and *Saturnia*, with the spider *Nephila edulis* as the outgroup. Bombycidae feedstocks such as *Bombyx mori* silk comprise heavy and light chain fibroins as well as P25 linked with disulphide bonds (Chevillard et al., 1986; Mita et al., 1994) in a 6:6:1 ratio (Inoue et al., 2000). In contrast, feedstocks of the Saturniidae such as *Antheraea yamamai*, *Actias luna*, *Attacus atlas* and *Saturnia pavonia* (Tanaka and Mizuno, 2001) comprise a homodimer (double heavy chain, H–H) protein mixture. As arthropods, spiders are very distantly related to the silkworms, yet by all accounts evolved silk production independently around 400 million years ago (Craig, 1997). Yet, the similar flow properties of their feedstocks thus represent an excellent example of convergent evolution (Craig, 1997; Holland et al., 2006).

**Fig. 1.**
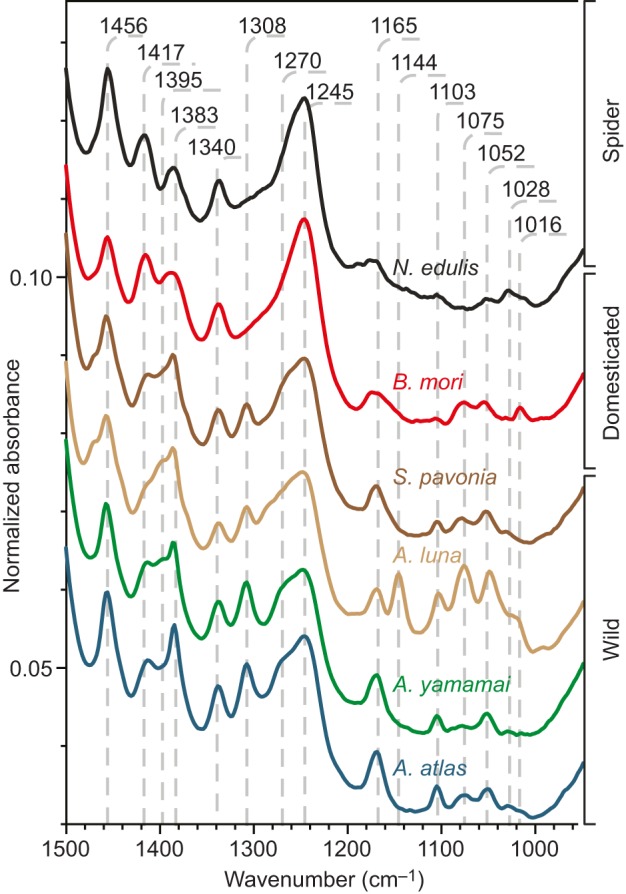
**Infrared spectra of unspun native silk feedstock.** Data are for domesticated silkworm silk (*Bombyx mori*), wild silkworm silk (*Attacus atlas*, *Antheraea yamamai*, *Actias luna*, *Saturnia pavonia*) and spider silk feedstock (*Nephila edulis* major ampullate). The 1700–1500 cm^−1^ region is not shown as little difference between species was observed. Infrared spectra were collected from feedstocks extracted directly from the animal and kept at a native concentration (∼22% dry weight).

Fig. 1 illustrates the distinctive features of native silk feedstock infrared spectra between 900 and 1500 cm^−1^ for silk from a variety of species. Table 1 indexes band assignments. Peaks between 1340 and 1456 cm^−1^ are commonly assigned to the vibration mode of residues (Barth, 2000). The strong 1383 cm^−1^ band associated with CH_2_ bending for wild silks (the top four curves in Fig. 1) suggests a higher proportion of long-chain residues in feedstocks from *B. mori* and *N. edulis* major ampullate silk glands. Another important distinction for wild silk feedstocks is the presence of the well-resolved 1308 cm^−1^ peak in the amide III region. Monitored by Rheo-IR (Boulet-Audet et al., 2014), this band vanishes under shear-induced denaturation (see supplementary material Fig. S1) and is absent from cocoon spectra. We have assigned this component to β-turns that are precursors to β-sheets formed after spinning (Bandekar and Krimm, 1980; Cai and Singh, 2004; Rousseau et al., 2006). The arginine–glycine–aspartic acid (RGD) residue pattern (Sukopp et al., 2002) is believed to procure a greater fibroblast proliferation rate to the wild silkworm *Antheraea mylitta* compared with domesticated *B. mori* silk (Minoura et al., 1995; Navarro et al., 2008). Hence, we hypothesized that RDG patterns might contribute to the wild silk-specific vibration mode at 1308 cm^−1^. The amide III shoulder at 1270 cm^−1^ results from α-helices (Cai and Singh, 2004; Krimm and Bandekar, 1986), and also appears stronger in wild silkworm silk feedstock. The neighbouring peak at 1245 cm^−1^ is commonly assigned to random coil secondary structures (Cai and Singh, 2004; Shao et al., 2005; Taddei and Monti, 2005; Yoshimizu and Asakura, 1990), and is strongest in the non-wild silks of *B. mori* and *N. edulis*. While present for all silk feedstocks, the peak at 1165 cm^−1^, associated with the stretching of the N–C_α_ bond, is clearly broader for non-wild species, suggesting a wider distribution of conformations.

**Table 1.**
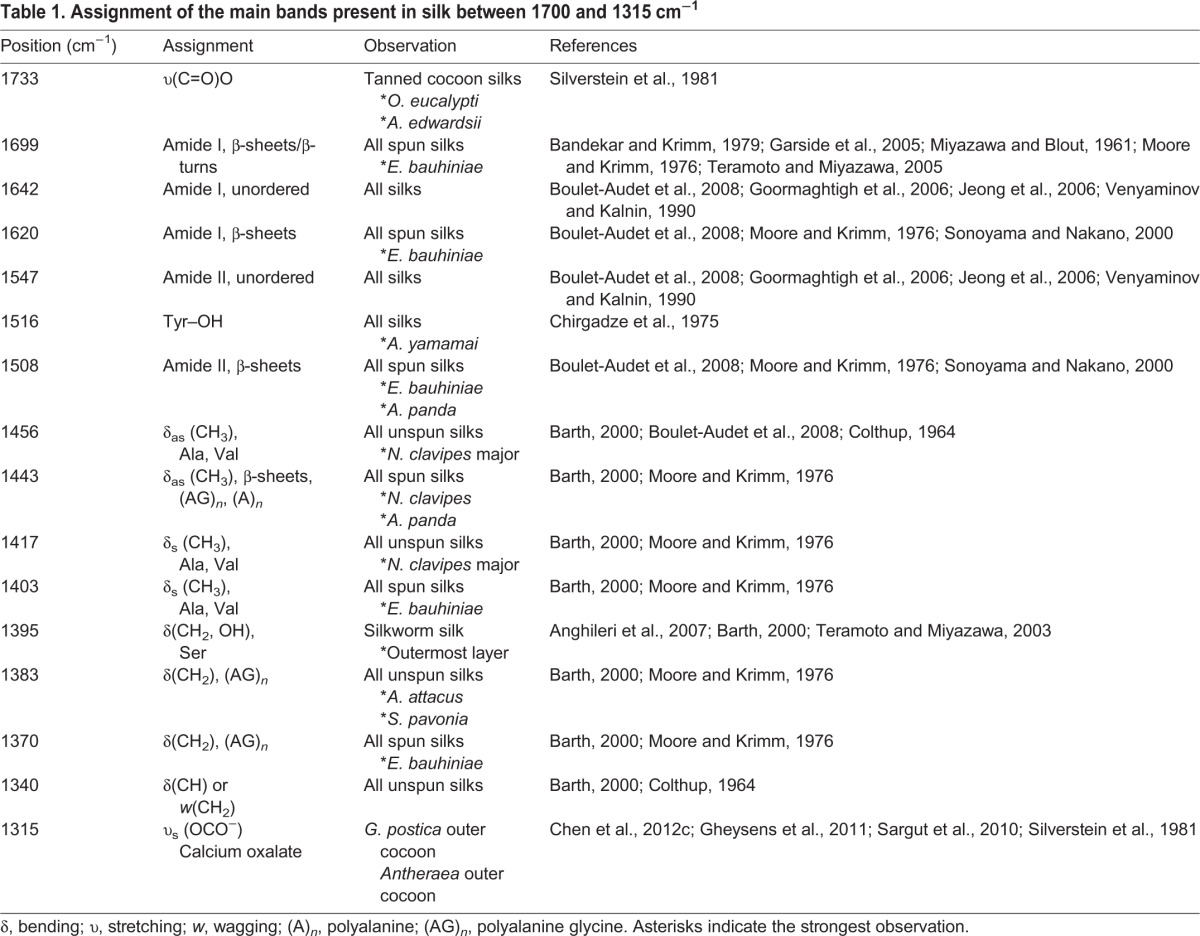
**Assignment of the main bands present in silk between 1700 and 1315 cm^−1^**

*Actias luna* is the only species probed that shows a well-resolved peak at 1144 cm^−1^. We speculatively assigned this distinct band to the C–O stretching of sericin-like components used as a binding resin/matrix, as this species produces a cocoon with low porosity and high density (Chen et al., 2012c). While this study focused on unprocessed silk, extracting the sericin for further analysis could help to clarify this speculative assignment.

The 1103 cm^−1^ band appeared on all spectra collected, although it was stronger in wild silkworm feedstocks. In the skeletal vibration region, this peak is likely to be caused by the C–C stretching of tyrosine aromatic rings, tryptophan or phenolic compounds (Andrus, 2006; Barth, 2000; Taddei and Monti, 2005). The adjacent component at 1075 cm^−1^ is present in all silk feedstocks (except sericin-free spider silk) and is also observed in pure sericin spectra, but is strongest in *A. luna*, thus reinforcing our previous assignment of the 1144 cm^−1^ peak for this species (Anghileri et al., 2007; Barth, 2000; Gupta et al., 1997). We also assigned the band at 1052 cm^−1^ to sericin C–O stretching, which is well resolved in most silkworm silks (Gupta et al., 1997; Taddei and Monti, 2005; Teramoto and Miyazawa, 2003).

### Cocoon silk spectral features

Our findings (above) show that silk feedstocks have clear spectral differences between species. Therefore, we must assume that the cocoons produced from these feedstocks would also show variability. Moreover, we would also expect this diversity to increase as construction introduces variables such as the larva's spinning behaviour, other silkworm secretions such as faeces, and exogenous materials such as tannins diffusing from leaves. Previous work has shown that the properties of silk cocoons vary between the innermost and outermost layers (Chen et al., 2012a). Thus, to examine these sources of chemical diversity, we compared the infrared spectra of the innermost and outermost layers of cocoons from 34 species of silkworm alongside the spectra of *N. edulis* dragline silk. Because of silk's molecular alignment, spectra will vary depending on the orientation of the fibres relative to the beam path (Boulet-Audet et al., 2008; Papadopoulos et al., 2007). For a fair comparison against cocoons without preferential orientation (Chen et al., 2012b), spider silk filaments were arranged in a similar random orientation order.

**Fig. 2.**
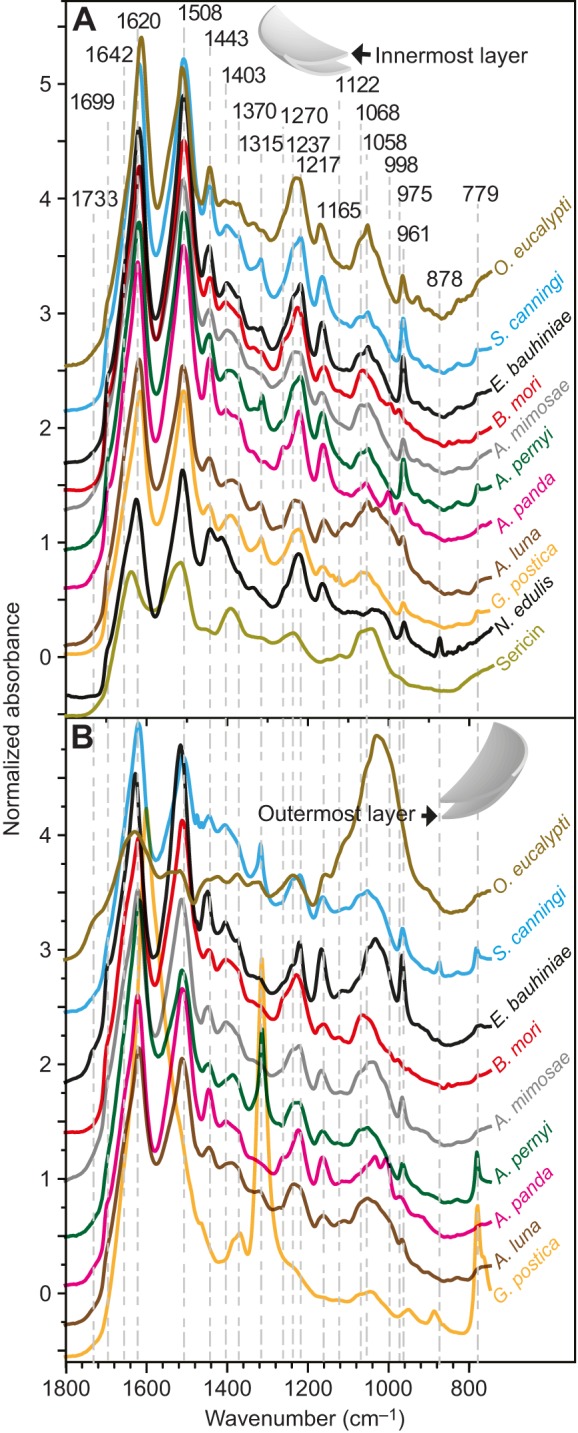
**Infrared spectra** **of the innermost and outermost layer of the cocoon of selected species.** (A) Infrared spectra of the innermost cocoon layer of key species, and *B. mori* sericin spectrum for comparison. (B) Outermost cocoon layer spectra.

Fig. 2A shows spectra acquired from the innermost part of the cocoons from selected distinctive species. While the primary constituent of these cocoons is still silk proteins, the cocoons' infrared signature differed substantially from that of their respective feedstocks. We assigned these differences to a number of causes: the water content is lower in cocoons, reducing the ratio of amide I to amide II height (1642/1508 cm^−1^); and precursor helical structures and random coils present in the feedstocks are converted via spinning into β-sheets, resulting in decreasing absorbance at 1642, 1547, 1308 and 1245 cm^−1^ and rising absorbance at 1699, 1620, 1508, 998 and 961 cm^−1^ (see Tables 1, 2). The relative absorbance of these β-sheet peaks can serve as an indicator of protein crystallinity. The peaks at 1699 and 1620 cm^−1^ in the amide I region are commonly used to determine the anti-parallel β-sheet content, but this overlaps with adjacent components from the fibroin and other compounds present in cocoons. In contrast, the low-frequency component at 961 cm^−1^ assigned to poly-alanine (A)*_n_* is much better resolved (Moore and Krimm, 1976; Papadopoulos et al., 2007; Taddei and Monti, 2005). This band also appears in the *N. edulis* dragline and most spectra of wild silk cocoons, particularly that of *Epiphora bauhiniae*. In contrast, some species like *B. mori* and *Anaphe panda* have two weaker peaks at 998 and 975 cm^−1^ as their β-sheets are constituted instead of poly(alanine–glycine) segments (Moore and Krimm, 1976; Taddei and Monti, 2005).

**Table 2.**
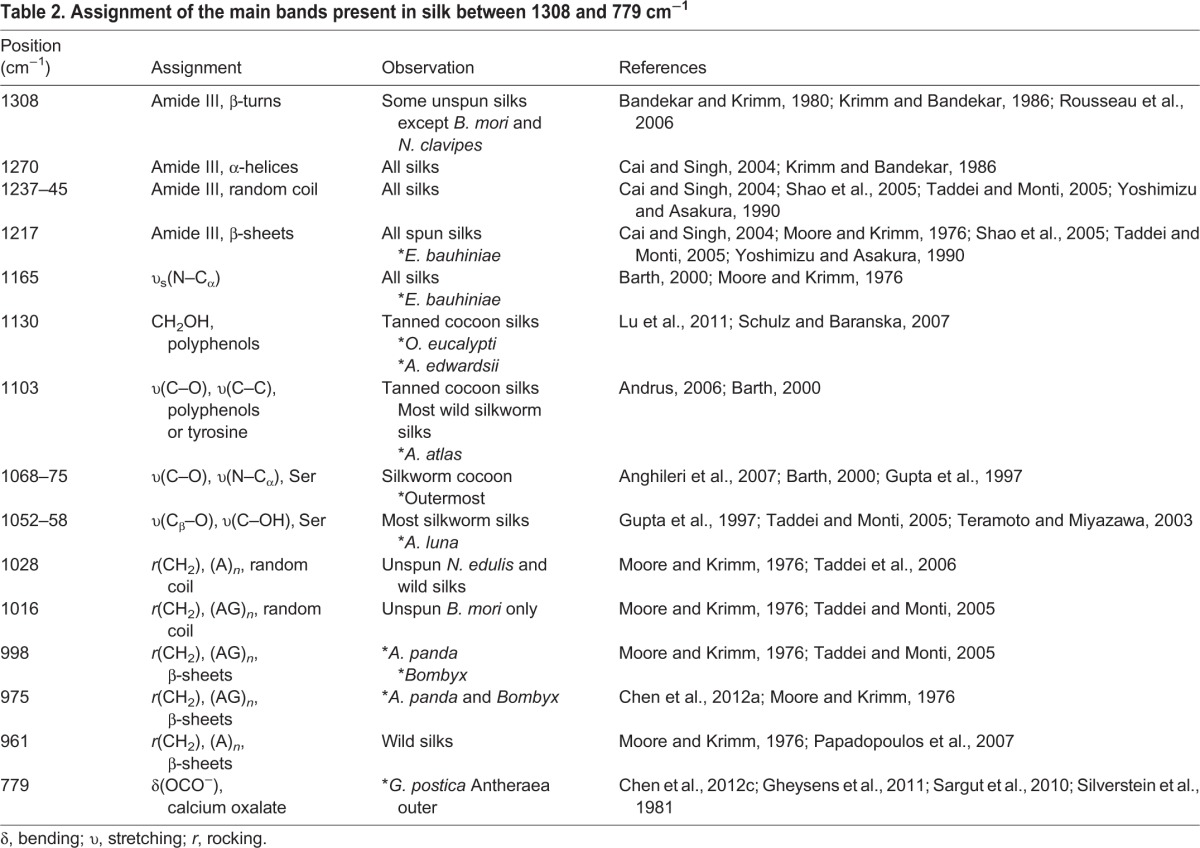
**Assignment of the main bands present in silk between 1308 and 779 cm^−1^**

Fig. 2B demonstrates that the distinctive spectral features observed in the innermost layer of the cocoons are even more prominent in the outermost layer. For example, there is a higher relative absorbance of bands between 1395 and 1058 cm^−1^, which is consistent with the greater amount of sericin in the outermost layer for species like *B. mori.* For comparison, a pure spectrum of sericin is included in Fig. 2A (Chen et al., 2012a). Another clear difference between the two layers is the amount of calcium oxalate [Ca(COO)_2_] crystals found embedded in the outermost layer of some species (Freddi et al., 1994, 1993; Gheysens et al., 2011; Takahashi et al., 1969; Teigler and Arnott, 1972b). Calcium oxalate vibration modes at 1315 and 779 cm^−1^ dominate the outermost layer of *Gonometa postica* cocoons yet are much weaker in the innermost layer. Another spectral contrast between layers was found in *Opodipthera eucalypti*, where the shoulder at 1733 cm^−1^ can be assigned to the carboxylic acid and the polyphenol hydroxyls around 1000 cm^−1^ (Andrus, 2006; Silverstein et al., 1981).

Thus, in summary, we established that it is possible to use structural and chemical markers to determine the type of crystallinity, the presence of sericin and calcium oxalate, and the polyphenol content in the measured cocoons.

### Between-species comparison of the chemical composition of cocoons

**Fig. 3.**
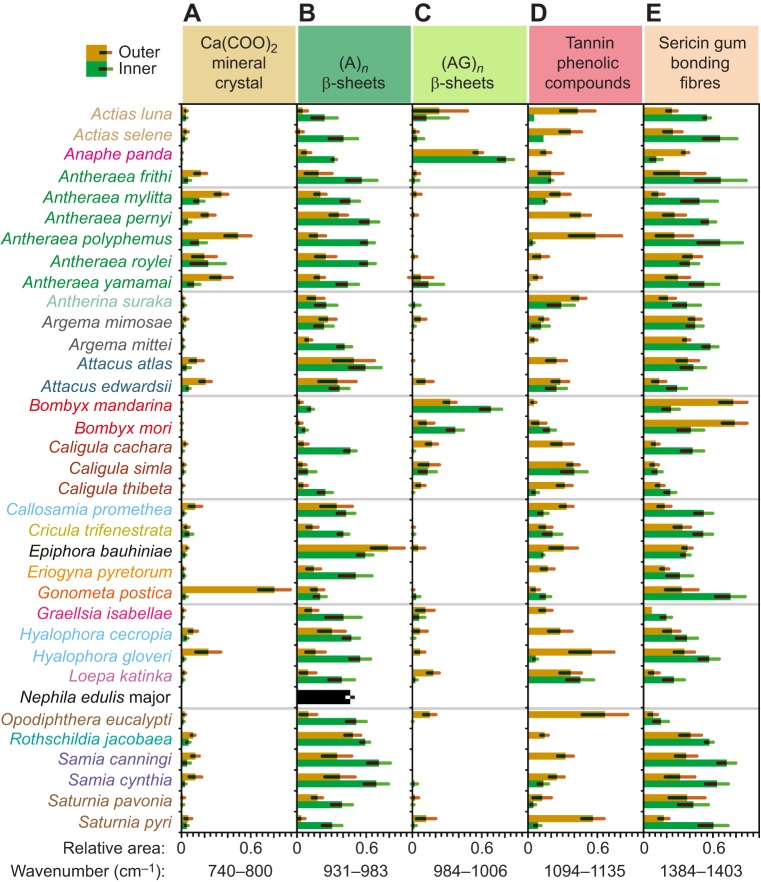
**Composition of cocoon outer and inner layers.** (A) Relative area of a band assigned to calcium oxalate (740–800 cm^−1^). (B) Relative area of the band associated with (A)*_n_* β-sheets (931–983 cm^−1^). (C) Relative area of the band assigned to (AG)*_n_* β-sheet (984–1006 cm^−1^). (D) Relative area of a band associated with tannins (1094–1135 cm^−1^). (E) Relative area of a sericin marker band (1384–1403 cm^−1^). The outermost and innermost layer values are shown (see key). The error bars represent the s.d. of the different observations (*N*>10). A value of 1 represents the highest area calculated and 0 represents the minimum measured.

For each spectrum collected, the interesting peaks identified above (see Fig. 2A,B) were integrated to estimate the relative content of calcium oxalate (1315 and 779 cm^−1^), β-sheet crystallinity (1699, 1620, 1508, 998 and 961 cm^−1^), the presence of tannin/phenolic compounds (1000 and 1733 cm^−1^) and the sericin resin/matrix (1395 and 1058 cm^−1^) (Fig. 3).

### Calcium oxalate mineral crystals

Calcium oxalate, also called raphide, forms highly toxic needle-like crystals, which can tear soft tissues and are thought to represent a plant defence mechanism (Arnott and Webb, 2000). Because no known metabolic pathways process calcium oxalate in silkworms, we assume that calcium oxalate presence in the cocoon is a result of the ingestion of leaves containing the compound and resultant excretion by the silkworm. While this may be the case for wild silkworms, it appears that artificial selection has changed the behaviour of the *B. mori* silkworm to prevent this excretion into the cocoon. Fig. 3A shows the relative intensity of the band at 779 cm^−1^ achieved by integrating the absorbance between 740 and 800 cm^−1^. This well-resolved band was used as a relative indicator of the amount of microscopic calcium oxalate monohydrate [Ca(COO)_2_] mineral crystals present in the cocoons (Chen et al., 2012b; Gheysens et al., 2011). Our ATR-IR results identified cocoons from *G. postica* as having the highest calcium oxalate content. The host plant of *G. postica*, *Acacia*, is also rich in calcium oxalate as a means to detoxify calcium ions (Franceschi and Nakata, 2005; Martin et al., 2012; Teigler and Arnott, 1972a). The high calcium oxalate content of *G. postica* and *Antheraea* genera cocoons measured is in agreement with previous reports and electron microscopy observations on these cocoons (Chen et al., 2012b; Gheysens et al., 2011). Cocoons from *Samia*, *Hylophora* and *Attacus* species also indicate the presence of calcium oxalate, but in lower proportions while other species measured have only minute amounts in their cocoons.

The presence of calcium oxalate in the cocoon is known to complicate the industrial reeling as it prevents the extraction of long lengths of fibre (Gheysens et al., 2011). Calcium oxalate is notoriously toxic to humans and responsible for kidney stone formation (Evan et al., 2007). The commonplace edetic acid (EDTA) treatment for dissolving kidney stones was found to be equally effective at demineralizing wild silk cocoons containing calcium oxalate crystals and enabling industrial processing (Gheysens et al., 2011). Thus, the ability to detect and quantify the amount of calcium oxalate present in a cocoon prior to processing may have industrial advantages in minimizing reagent use or in selecting low mineral content cocoons in the first place.

### β-Sheet crystallinity

X-ray scattering initially showed the presence of β-sheet nanocrystals inside silk fibres (Warwicker, 1954). Conveniently, polyalanine (A)*_n_* and polyalanine glycine (AG)*_n_* β-sheet structures also give distinctive peaks in silk infrared spectra, indicative of the degree of crystallinity present, and by extension may relate to mechanical properties (Boulet-Audet et al., 2008; Moore and Krimm, 1976; Porter et al., 2005; Sonoyama and Nakano, 2000). Using the integrated absorbance of (A)*_n_* antiparallel β-sheets peaking at 931–983 cm^−1^, Fig. 3B shows the relative (A)*_n_* β-sheet content across the species tested. Our results suggest that *E. bauhiniae* has the highest degree of crystallinity amongst all the (A)*_n_*-containing silks measured, followed by species from the *Samia*, *Antheraea* and *Attacus* genera. For most species, the (A)*_n_* β-sheet content appears greater in the innermost layer, probably due to non-fibroin compounds contributing to the infrared signal more on the outermost layer. Spider silk dragline from *N. edulis* appears to have a comparable (A)*_n_* β-sheet crystallinity to that of most silkworm silks measured. *Gonometa*, *Argema* and *Caligula* genera seem to have the lowest (A)*_n_* β-sheet content amongst all the species studied. Integrating the region between 984 and 1006 cm^−1^ quantified the contribution of the (AG)*_n_* peaks at 975 and 998 cm^−1^ while excluding the (A)*_n_* β-sheet peak at 961 cm^−1^. Only three species appear to have (AG)*_n_* repetitive segments, *B. mori*, *Bombyx mandarina* and *A. panda* (see Fig. 3C). Unlike Bombycidae and Noctuidae families, none of the Saturniidae cocoons displayed peaks associated with the (AG)*_n_* structure (Moore and Krimm, 1976; Taddei and Monti, 2005). This fundamental distinction could be related to their appurtenance to different taxonomic families (see below).

### Tannins and phenolic compounds

Wild silkworms naturally secrete some phenolic compounds in their silk (Brunet and Coles, 1974), but our results suggest that additional hydroxyl-containing compounds, such as polyphenols, could come from exogenous sources. By integrating the absorbance between 1035 and 1094 cm^−1^, the relative amount of these molecules can be estimated. Fig. 3D shows that a few species had phenolic compounds, located mainly on the outside of the cocoon, including *O. eucalypti*, *Saturnia pyri*, *Hyalophora gloveri*, *Attacus edwardsii*, *Antheraea polyphemus* and *A. luna*. This finding agrees with the hypothesis that leaves incorporated by the silkworm into the cocoon structure leech water-soluble plant polyphenols when wet. In contrast, species that do not integrate leaves into their cocoons, such as *A. mylitta* and *A. atlas*, showed low phenolic compound parameter scores.

### Sericin protein gum

Sericin proteins are essential to cocoon construction as they are used to bond fibres together (Chen et al., 2012a). The amount of sericin present in a cocoon can be inferred from the absorption bands between 1384 and 1403 cm^−1^ associated with the amino acid serine, which is present in high quantities in sericin but not in fibroin (Teramoto and Miyazawa, 2003). Fig. 3E suggests that *Bombyx* genus silks have the most sericin along with *Actias*, *Antheraea*, *Saturnia* and *Samia* genera silks. Our results indicate that there is less sericin in the coats of the high-porosity cocoons of species such as *Cricula trifenestrata*, *Graellsia* *isabellae* and *Loepa katinka* (Chen et al., 2012b). Differences in sericin abundance between the innermost and outermost layers of the cocoons tested indicate that *B. mori* cocoons have more sericin in the outermost layer, consistent with previous findings (Chen et al., 2012a). However, because of the additional mineral and phenolic components of the wild silks, it is challenging to interpret the distribution reliably in the other silks tested.

### Classification of silk species

Our results show that the integration of infrared spectra bands assigned to individual compounds can provide select windows into a silk cocoon's chemical composition. However, single variable analysis exploits only a small fraction of the information contained within the spectra with thousands of data points. In contrast, multivariable analysis is far more powerful for classifying and discriminating samples. Hence, we first performed a principal component analysis (PCA) (Pearson, 1901) to reduce the number of variables while retaining most of the variability. The first principal component (PC) expresses the largest variance between samples. The PC scores, indicating the relative importance of these PC for each spectrum, were subsequently used for the linear discrimination analysis (LDA) to model the differences between species with a set of factor coefficients and scores.

The LDA scores were able to discriminate broadly between wild and domesticated silks as well as spider silk. Of the 25 measurements selected randomly for validation, the method identified the correct species for 100% of the ‘unknown spectra’ (see Materials and methods). Supplementary material Fig. S2 shows the tree generated from the LDA scores using hierarchical clustering analysis (HCA). However, as previously noted, once the silk has been spun into the cocoon structure, even more variables are introduced, and thus our multivariate approach becomes even more powerful. The multivariate analysis had an identification hit rate of 70% for species and 75% for genus, tested using the randomly selected validation group of 200 ‘unknown spectra’ (see Materials and methods).

**Fig. 4.**
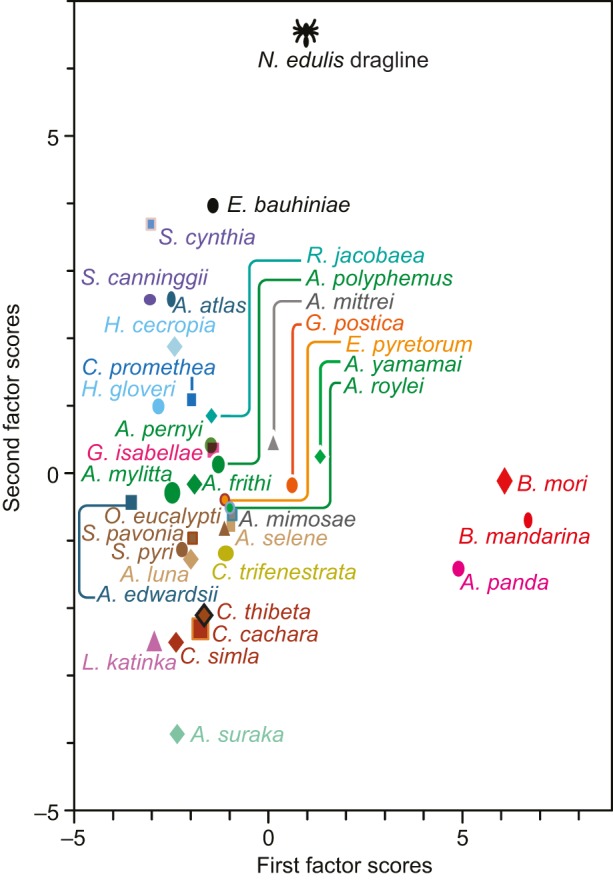
**Factor scores of the cocoon spectra.** The first and second factor scores contribute to 62% of species discrimination of the linear discriminant analysis.

Our initial multivariate analysis of cocoon diversity is summarized in Fig. 4, which highlights the values of the first and second factor scores calculated from the cocoon spectra. The primary cluster encompasses most silks from wild silkworm species with *Antheraea* silks near its centroid (green markers). *Antherina suraka*, *L. katinka*, *E. bauhiniae* and *Samia cynthia* silks appear in the periphery of the cluster, suggesting a greater dissimilarity with the average of the measured silks. Clearly discriminated species outside this cluster such as *A. panda*, *B. mori* and *B. mandarina* appear as outliers. The *N. edulis* spider dragline silk is also outside the primary cluster, and easily discriminated from silkworm cocoons with the second factor scores. Notably, our LDA implies that *E. bauhiniae* is the silkworm species producing the closest silk to the *N. edulis* dragline. However, more species from other families would need to be studied to identify which of the thousands of silkworm species spins ‘spider silk’. To develop this analysis further and begin to draw quantitative links between species, our HCA used the scores of the 10 most important factors to group these species according to their similarity.

#### Group 1: *Caligula*, *Saturnia* and *Actias*

**Fig. 5.**
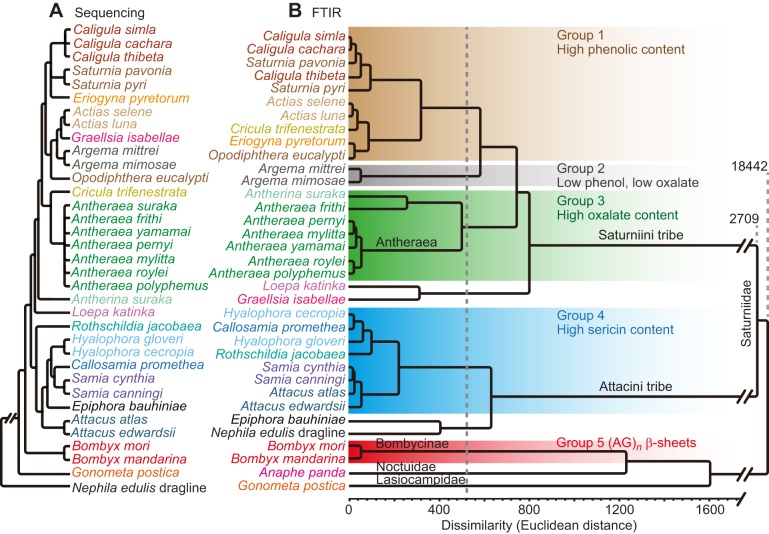
**Classification of silk species.** (A) Cladogram generated from the phylogenetic analysis of Regier et al. (2005, 2008a,b, 2002; see also Chen et al., 2012c). (B) Ultrametric tree generated from the hierarchical clustering analysis of cocoon infrared spectra LDA factor scores. Species with a Euclidean distance smaller than 525 were grouped together. FTIR, Fourier transform infrared spectroscopy.

Group 1 (see Fig. 5B) encompasses *Caligula*, *Saturnia* and *Actias* genera together with *O. eucalypti* and *C. trifenestrata*. As most species from this group had high absorbance between 1094 and 1135 cm^−1^ (Fig. 3D), this result suggests that these species were grouped together partly based on their high phenolic content. Except for *C. trifenestrata*, these species’ cocoons appeared substantially tanned with a dark brown coloration (Chen et al., 2012c). Also, these species do not present calcium oxalate crystals on their surface (Chen et al., 2012c), as confirmed in Fig. 3A. Group 1 also appears to have a lower β-sheet content than the other groups.

#### Group 2: *Argema*

Group 2 contains the *Argema* genus. Unlike cocoons from group 1, they do not appear to have a high phenolic content or calcium oxalate, and although largely comparable to neighbouring groups these two factors could explain the large Euclidean distance from group 1.

#### Group 3: *Antheraea*

From our classification, it appears that *Antheraea* silks all have small Euclidean distances relative to one another and as such they were all grouped together with *A. suraka* in group 3. Previous studies based on morphological feature classification argued that *A. suraka* could be more closely related to the African Bunaeini tribe than other species of the Saturniini (Oberprieler, 1997). The comparable Euclidean distance between *A. suraka* and *Antheraea frithi* weakens this hypothesis. As *Antheraea* is the genus with the most calcium oxalate (Fig. 3A), the LDA method could have regrouped these silks mainly on mineral content. However, *A. suraka* and *A. frithi* show less absorption between 740 and 800 cm^−1^ (calcium oxalate) and are more distant from the other species of this group. In addition, group 3 has weaker phenolic compound bands than group 1 and yet has an average amount of sericin. The next closest species to these groups are *L. katinka* and *G. isabellae*, which both present a low sericin content and high porosity according to other studies (Chen et al., 2012b,c).

#### Group 4: *Attacus* and *Samia*

Much more distant are the species classified in group 4, including *Samia*, *Hyalophora* and *Attacus* genera along with *Callosamia promethea*. When compared with previous reports, the morphology of the cocoons classified together in group 4 appears characteristic as the innermost layers are much more compact than their outer layers (Chen et al., 2012c). This morphological difference could explain the higher amount of sericin measured in the innermost layer (Fig. 3E). Adding to the composition variation between the innermost and outermost layers, these cocoons have an intermediate content of (A)*_n_* β-sheets, sericin and tannin when compared with those from groups 1 and 3. Branching from group 4, *E. bauhiniae* has the largest amount of (A)*_n_* β-sheets, little phenolic compounds, no calcium oxalate and little sericin.

#### Group 5: *Bombyx*

The LDA placed *B. mori* and *B. mandarina* silks into a distant group. Even though *B. mandarina* appears to have more (AG)*_n_* β-sheets than *B.* *mori* (Fig. 3C), the difference between their spectra is subtle in comparison with the other species presented. This result suggests that the artificial selection of *B. mori* might have played a lesser role than natural selection in differentiating this species from other Lepidoptera families.

#### Silk from other superfamilies

While still very distant, *A. panda* had the smallest Euclidean distance to *Bombyx*. Our results suggest that *A. panda* also has (AG)*_n_* β-sheets, sericin and no calcium oxalate. Testing more silks from these two families would confirm whether these silks share the same spectral features. Social spinning behaviour is another interesting characteristic of *A. panda*, which partners with many other worms to build a communal cocoon nest (Mbahin et al., 2007). The cocoon's structure does not depend on the silk quality of a single individual, resulting in different natural selection constraints. Also from another superfamily (Lasocampiadae), *G. postica* silk is very distinct from all other species studied. The innermost layer appears closer to Saturniidae silks (Fig. 2), whereas the major difference between their outer layer is likely to come from the large amount of calcium oxalate present.

### Comparison of ATR-IR and phylogenetic trees

The ultrametric tree generated from the infrared spectra (see Fig. 5A) was compared with the phylogenetic tree built from the sequencing of a few protein-coding nuclear genes by Regier et al. (2008a,b, 2002; see also Chen et al., 2012c). The genes selected to construct this phylogeny produce proteins other than silk, with various enzymatic functions such as carbamoylphosphate synthetase, aspartate transcarbamylase, dihydroorotase (Moulton and Wiegmann, 2004), dopa decarboxylase (Fang et al., 1997), enolase (Farrell et al., 2001) and wingless (Brower and DeSalle, 1998). Although a quantitative comparison between an ultrametric and a unitless tree is not possible, they are strikingly similar, except for few species.

## DISCUSSION

By assessing the diversity of wild silks, this study compared the biochemical composition of native silk feedstock from six species and silk cocoons from 34 species using infrared spectroscopy and multivariate analysis. For unspun native silk feedstocks, we identified new spectral markers unique to wild silkworm silks, which we assigned to β-turn secondary structures. The hierarchical clustering of the feedstocks also profiled the dissimilarity of Saturniidae silks to the silks of Bombycidae and spiders.

Collecting spectra from silkworm cocoons provided information not only on the spun fibre but also on the non-protein chemical content and distribution across the layers. The specific infrared bands revealed the relative content of sericin, calcium oxalate, phenolic compounds, (A)*_n_* and (AG)*_n_* β-sheets. The multivariate analysis also permitted the hierarchical classification of 35 species (including one spider silk) into groups based on their chemical composition. This analysis revealed the presence of interesting outlier species with very dissimilar spectra, which could manifest as distinctive mechanical or chemical properties. Amongst these outliers were *G. postica* cocoons, which had the highest calcium oxalate of all species measured. Furthermore, the species with the most β-sheets, *E. bauhiniae*, also appeared to have the closest chemical composition to *N. edulis* spider silk dragline. The *Bombyx* genus stood out from all other species measured, representing an outlier group. Consequently, using *B. mori* as the model species for silk studies could lead to conclusions that are not applicable to all types of silks. Although our sampling had a bias towards Saturniidae silk, *Antheraea* silks were found to have median PC scores, suggesting that *Antheraea* silks are more representative of silk biodiversity. Not only did the multivariate analysis have a species identification hit rate of 70% but also the ultrametric trees were created from the infrared spectra.

Our analysis thus suggests a relationship between non-silk coding nuclear genes selected by Regier et al*.* and the silkworm cocoon's overall biochemical composition (Regier et al., 2005, 2008a,b, 2002). Such a link implies that infrared spectra could be used as a proxy for the phylogenetic classification of species. Despite huge similarities between these trees, a few silk species were classified differently under these two approaches. This difference could be the result of non-protein-based variation such as temperature or humidity or the incorporation of exogenous material into the cocoons. For instance, *C. trifenestrata* was expected to be closer to the *Antheraea* silks rather than classified into group 1. The difference could be due to the fact that *C. trifenestrata* lives in an environment with a warm climate, requiring more ventilation than *Antheraea* silk cocoons found in colder regions (Kakati and Chutia, 2009). Interestingly, *G. isabellae* silk should have been very similar to *Actias* silks, but was classified by our analysis outside group 1 along with *L. katinka*. Their separate classification could result from the high concentration of tannins measured in the cocoons of these species. Oberprieler and Nassig (1994) suggested that these species might have been misclassified, and our study strengthens the hypothesis that *Graellsia* and *Actias* are two distinct genera. As expected, *E. bauhiniae* was classified in the Attacini tribe but is rather distant from the other species of group 4, most likely because of its higher (A)*_n_* β-sheet crystallinity content. In summary, despite minor differences in the classifications, our method represents a powerful but straightforward hierarchical classification tool to help resolve some of the ambiguity in the relationships of Lepidoptera species.

Because of the intense selection pressure on this vital biological structure, we believe silk cocoons represent a model for the phylogenetic analysis of all silkmoth species. This untapped proxy method not only adds to more traditional gene and protein sequencing but is also less time consuming and cheaper than, for example, protein sequencing. As silk cocoons are commonly part of entomology collections spanning hundreds of years of sampling, they can be readily sourced and rapidly tested in a non-destructive manner. Such powerful longitudinal studies could shed light on silkmoth evolution and ecology by helping to resolve some of the relationship ambiguities of Lepidoptera species. Furthermore, with the advent of affordable handheld IR instruments, our approach could also allow such analysis to take place in the field. Thus, combining ATR-IR with multivariate analysis could aid in unravelling the evolution and biodiversity of silk-producing species as well as inform us regarding which species is best suited to a particular industrial application.

## MATERIALS AND METHODS

### Native silk feedstock preparation

All wild silkworm eggs were purchased from Worldwide Butterflies (WWB, Dorset, UK). *Actias luna*, *A. yamamai*, *A. atlas* and *S. pavionia* were fed with walnut (*Juglans regia*), hawthorn (*Crataegus monogyna*), privet (*Ligustrum vulgare*) and hawthorn (*C. monogyna*), respectively. When larvae started spinning their cocoon, native silk feedstocks were extracted from the silk glands of last instar silkworms. Final instar *B. mori* worms were fed with white mulberry leaves (*Morus alba*). *Nephila edulis* major ampullate glands and dragline were extracted from mature female spiders fed with *Drosophila* spp. and *Caliphora* spp. and reared in-house under controlled temperature and humidity as described elsewhere (Holland et al., 2006).

### Silk cocoon preparation

**Fig. 6.**
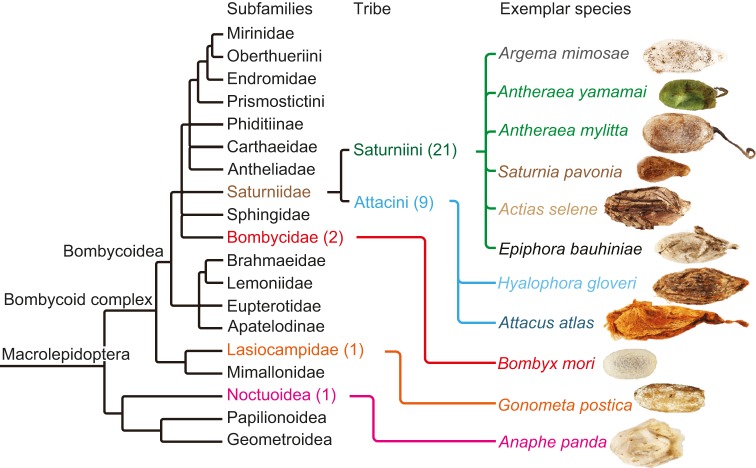
**Summary of higher-level relationships of the subfamilies related to species studies adapted from** **Regier et al. (2008a****).** Numbers in parentheses represent the number of species measured in the subfamilies. The images on the right are the cocoons of the species measured.

We analysed cocoons from 34 species across the Lepidoptera. The superfamilies Saturniini and Attacini are highlighted in Fig. 6. The International Centre of Insect Physiology and Ecology (icipe; African Insect Science for Food and Health) in Kenya provided *G. postica* silkworm cocoons. The other cocoon species were purchased from WWB; the species chosen cover four Lepidopteran families. At least four, 3.5 mm round cocoon discs were cut from each cocoon using a plier punch for analysis by infrared spectroscopy.

### Spectral acquisition and treatment

A Golden Gate single bounce diamond ATR accessory (Specac Ltd, London, UK) coupled to a Nicolet 6700 FTIR spectrometer equipped with a MCT nitrogen cooled detector (Thermo Scientific, Madison, WI, USA) was used for spectra collection. Spectra acquisition was performed at a 4 cm^−1^ resolution from 500 to 6000 cm^−1^, averaging 32–64 scans at a 5.06 cm s^−1^ mirror speed. Although the fibres in a cocoon sample are randomly oriented, spectra were collected with the infrared beam polarized perpendicular to the plane of incidence (*s*) with a zinc selenide holographic wire grid polarizer (Thermo Scientific). The ATR diamond's internal reflection element (IRE) had a refractive index of 2.417 and an angle of incidence of 45 deg. For this configuration, the evanescent wave emerging out of the IRE could probe around 1.2 μm deep into the sample, the penetration varying with the wavelength (Harrick, 1967). The liquid state of native feedstock spectra ensured a good contact with the IRE for data collection. As the cocoons have an inherent roughness greater than tens of micrometres, an anvil was used to press on the cocoon discs to ensure a good contact with the IRE. For acquisition consistency, the pressure applied on cocoon discs was kept to the minimum necessary to obtain an absorbance of 0.1 for the amide II band. By aiming to keep the absolute absorbance consistent, the anomalous dispersion of the refractive index was therefore comparable for each spectrum collected (Boulet-Audet et al., 2010). Before each measurement, the crystal was cleaned with tissue and demineralized water before a new background was acquired. This method helped to compensate for the detector's signal fluctuations as well as preventing contamination between measurements. The innermost and outermost layers of these discs were measured by collecting at least 18 distinct spectra from each species for a total of 1185 spectra across the 35 species studied. This spectroscopic study thus encompasses the largest number of wild silk types to date.

### Data pre-processing

Spectral operations were performed using OMNIC 7.3 (Thermo Scientific) using a custom-written VBA code. An offset was first subtracted from all spectra as calculated from the average of the region from 1950 to 1900 cm^−1^. Spectra were then normalized using the integrated absorbance from 1900 to 800 cm^−1^ to compensate for absolute signal variations incurred by differing cocoon contact with the IRE. For the single component analysis, the relative area of each peak integrated was calculated by subtracting a linear baseline between the interval limits from the integrated absorbance.

### Multivariate analysis and dendrogram generation

Despite high spectral reproducibility, the absolute absorbance values vary between measurements depending on the contact between the porous cocoon and the IRE. To discriminate silks based on their spectral line shape and peak position rather than absolute absorbance values, the multivariate analysis was performed on the first derivative of the spectra. The second derivative is as effective, but enhances the noise further (Kansiz et al., 1999). Mid-infrared spectra contained 2853 variables, but were not all interdependent as a single compound spectral line often contributed in different regions simultaneously. To reduce the number of variables while preserving most of the dataset variability, a Pearson PCA (Pearson, 1901) was performed using XLSTAT (Addinsoft, Paris, France). Keeping only 10 PCs still preserved 90% of native silk feedstock spectral variability, while selecting the first 40 PCs still accounted for 86% of cocoon spectral variability.

A LDA (Fisher, 1936; Yang et al., 2005) was performed on the PC scores to find a linear combination of features that separate infrared spectra from silks of different species; 27 of 52 native silk spectra and 962 of the 1162 cocoon spectra were randomly selected for the training (estimation) group to construct the discrimination function. The remaining 25 native silk and 200 cocoon spectra were used to validate the discrimination function. The LDA achieved a hit rate of 100% for native silk and 70% for cocoon spectra while assigning 75% to the correct genus.

The LDA factor centroid scores were subsequently used to calculate the Euclidean distance between each species for Ward's HCA (Mariey et al., 2001; Ward, 1963). This method minimizes the total variance within clusters starting from singleton clusters (one species per cluster) in a top-down approach. The resulting HCA dendrograms were subsequently compared with the phylogenic tree dendrogram built from genetic data (Chen et al., 2012c; Regier et al., 2005, 2008a,b, 2002).

Supplementary material Fig. S1 shows the infrared spectra before and after shear-induced denaturation of *B. mori* and *A. atlas* along with their corresponding difference spectrum. Supplementary material Fig. S2 shows the ultrametric tree generated from the infrared spectra of native feedstock primary canonical functions.

Supplementary material Fig. S2 shows the tree generated from the infrared spectra of native feedstock main canonical functions using HCA. Sharing common spectral features such as the 961, 1103 and 1308 cm^-1^, feedstocks from *A. luna*, *S. pavonia* and *A. atlas* are more closely related. This result corroborates the fact that these four species are from the same Saturniidae arthropod superfamily. Although much more distant, the closest to these species is the silkworm silk *B. mori* feedstock as it is also a silkworm silk feedstock containing sericin proteins. With fewer types of fibroins and no sericin, spider silk feedstock infrared spectra are very distinct. Relative to the silkworm feedstock's dissimilarity, the spider silk feedstock tested has a much greater Euclidean distance.
